# Bi‐allelic pathogenic variants in *PABPC1L* cause oocyte maturation arrest and female infertility

**DOI:** 10.15252/emmm.202217177

**Published:** 2023-04-13

**Authors:** Weijie Wang, Jing Guo, Juanzi Shi, Qun Li, Biaobang Chen, Zhiqi Pan, Ronggui Qu, Jing Fu, Rong Shi, Xia Xue, Jian Mu, Zhihua Zhang, Tianyu Wu, Wenjing Wang, Lin Zhao, Qiaoli Li, Lin He, Xiaoxi Sun, Qing Sang, Ge Lin, Lei Wang

**Affiliations:** ^1^ Institute of Pediatrics, Children’s Hospital of Fudan University, the Shanghai Key Laboratory of Medical Epigenetics, the Institutes of Biomedical Sciences, the State Key Laboratory of Genetic Engineering, Fudan University Shanghai China; ^2^ Clinical Research Center for Reproduction and Genetics in Hunan Province, Reproductive and Genetic Hospital of CITIC‐Xiangya Changsha China; ^3^ Reproductive Medicine Center, Shaanxi Maternal and Child Care Service Center Xi'an China; ^4^ Human Phenome Institute, Fudan University Shanghai China; ^5^ NHC Key Lab of Reproduction Regulation (Shanghai Institute for Biomedical and Pharmaceutical Technologies) Fudan University Shanghai China; ^6^ Shanghai Ji Ai Genetics and IVF Institute, Obstetrics and Gynecology Hospital Fudan University Shanghai China; ^7^ Bio‐X Center, Key Laboratory for the Genetics of Developmental and Neuropsychiatric Disorders, Ministry of Education Shanghai Jiao Tong University Shanghai China

**Keywords:** female infertility, Mos‐MAPK, mRNA translational activation, oocyte maturation arrest, *PABPC1L*, Genetics, Gene Therapy & Genetic Disease, Urogenital System

## Abstract

Oocyte maturation arrest is one of the important causes of female infertility, but the genetic factors remain largely unknown. *PABPC1L*, a predominant poly(A)‐binding protein in *Xenopus*, mouse, and human oocytes and early embryos prior to zygotic genome activation, plays a key role in translational activation of maternal mRNAs. Here, we identified compound heterozygous and homozygous variants in *PABPC1L* that are responsible for female infertility mainly characterized by oocyte maturation arrest in five individuals. *In vitro* studies demonstrated that these variants resulted in truncated proteins, reduced protein abundance, altered cytoplasmic localization, and reduced mRNA translational activation by affecting the binding of PABPC1L to mRNA. *In vivo*, three strains of *Pabpc1l* knock‐in (KI) female mice were infertile. RNA‐sequencing analysis showed abnormal activation of the Mos‐MAPK pathway in the zygotes of KI mice. Finally, we activated this pathway in mouse zygotes by injecting human *MOS* mRNA, and this mimicked the phenotype of KI mice. Our findings reveal the important roles of PABPC1L in human oocyte maturation and add a genetic potential candidate gene to be screened for causes of infertility.

## Graphical abstract

Seven rare variants in *PABPC1L* were identified in five infertile females mainly characterized by oocyte maturation arrest followed a recessive inheritance pattern. *In vitro* and *in vivo* functional studies confirmed the destructive effects and pathogenicity of *PABPC1L* variants.

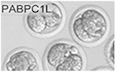

**The paper explained**

**Problem**

Oocyte maturation arrest is one of the common factors leading to repeated failures of assisted reproduction and female infertility. Studies have confirmed that genetic factors play a crucial role in oocyte maturation arrest, including *TUBB8*, *PATL2*, and *TRIP13*, but the underlying genetic factors for most affected individuals are still unknown.

**Results**

We identified compound heterozygous and homozygous pathogenic variants in *PABPC1L* that are responsible for female infertility mainly characterized by oocyte maturation arrest in five individuals from four independent families. *PABPC1L* encodes a poly(A)‐binding protein that plays a key role in translational activation of maternal mRNAs. *In vitro* studies demonstrated that pathogenic variants resulted in the functional impairment on PABPC1L, including truncated proteins, reduced protein abundance, altered cytoplasmic localization, and reduced mRNA translational activation by affecting the binding of PABPC1L to mRNA. *In vivo*, we constructed three strains of *Pabpc1l* KI mice corresponding to patient‐derived variants and found that female mice were infertile due to abnormal activation of the Mos‐MAPK pathway in zygotes. These results demonstrate the destructive effects and pathogenicity of the *PABPC1L* variants identified in affected individuals both *in vitro* and *in vivo*.

**Impact**

We identified and verified bi‐allelic pathogenic variants in *PABPC1L* that cause oocyte maturation arrest and female infertility. Our findings provide direct evidence for the important role of PABPC1L in human oocyte maturation and thus add a new marker for the genetic diagnosis of infertility patients in the clinic.

## Introduction

Mammalian oocyte maturation involves two successive meiotic divisions, and these are accompanied by a series of molecular events, including germinal vesicle breakdown (GVBD), spindle assembly, extrusion of the first polar body (PB1), and finally arrest at meiotic metaphase II (MII) (Mihajlovic & FitzHarris, 2018). Only fully mature MII oocytes can be recognized and fertilized by sperm and undergo early embryonic development (Plachot & Mandelbaum, 1990). Oocyte maturation is therefore a prerequisite for successful human reproduction. In clinical practice, oocyte maturation arrest is a common cause of recurrent failure of assisted reproductive technology (ART), including *in vitro* fertilization (IVF), and intracytoplasmic sperm injection (ICSI), and such failure is characterized by the repeated production of immature oocytes with resulting infertility (Huang *et al*, 2018). Four types of maturation arrest have been described, namely, germinal vesicle (GV) arrest, metaphase I (MI) arrest, MII arrest, and mixed arrest (oocyte arrest at more than one stage) (Beall *et al*, 2010). In 2016, we found that pathogenic variants in the primate‐specific gene *TUBB8* (MIM: 616768) cause human oocyte MI arrest by impairing oocyte spindle assembly (Feng *et al*, 2016). Subsequently, studies have shown that pathogenic variants in *PATL2* (MIM: 614661) and *TRIP13* (MIM: 604507) cause oocyte GV arrest and MI arrest, respectively (Maddirevula *et al*, 2017; Chen *et al*, 2017b; Zhang *et al*, 2020). Zona pellucida is an extracellular glycoprotein matrix composed of ZP1, ZP2, ZP3, and ZP4, which plays a vital role in oocyte maturation. Studies have reported that the variants in *ZP1*(MIM: 195000), *ZP2* (MIM: 182888), and *ZP3* (MIM: 182889) affect the formation of zona pellucida and result in female infertility (Huang *et al*, 2014; Liu *et al*, 2017; Dai *et al*, 2019), but there is no convincing evidence to prove that *ZP4* (MIM: 613514) variant responsible for female infertility. Pathogenic variants in *TUBB8*, *PATL2*, and *TRIP13* account for around 10% of infertility patients with oocyte maturation arrest (Feng *et al*, 2016; Huang *et al*, 2017; Maddirevula *et al*, 2017; Chen *et al*, 2017a, 2017b, 2019; Wang *et al*, 2018; Wu *et al*, 2019; Liu *et al*, 2020; Zhang *et al*, 2020; Cao *et al*, 2021; Yang *et al*, 2021; Yao *et al*, 2022). However, the genetic basis and the mechanisms involved in the majority of affected individuals remain unknown.

Oocyte maturation requires poly(A) tail‐modulated translational activation of dormant maternal mRNAs stored in the cytoplasm (Gebauer *et al*, 1994; Stebbins‐Boaz *et al*, 1996; Mendez *et al*, 2000). A specialized PABP (poly(A)‐binding protein) called *PABPC1L* (poly(A)‐binding protein cytoplasmic 1‐like) plays a key role in these processes. *PABPC1L*, also known as *EPAB* (embryonic poly(A)‐binding protein), was originally identified in *Xenopus* (Voeltz *et al*, 2001) and was found to be essential for *Xenopus* oocyte maturation (Friend *et al*, 2012). During *Xenopus* oocyte maturation, PABPC1L binds to the elongated poly(A) tail to stabilize polyadenylated mRNAs (Voeltz *et al*, 2001; Kim & Richter, 2007). Meanwhile, poly(A)‐associated PABPC1L promotes the dissociation of the translational suppressor Maskin from eIF4E and establishes eIF4G–eIF4E interactions for the initiation of translation (Cao & Richter, 2002). In mice, *Pabpc1l* is expressed in both immature GV and MI oocytes and mature MII oocytes, as well as in one‐cell and two‐cell embryos, and it is replaced by a somatic PABP (*Pabpc1*) after zygotic genome activation (ZGA) (Seli *et al*, 2005). *Pabpc1l* ^−/−^ female mice are infertile and unable to generate mature oocytes due to impaired translational activation of maternal mRNAs, including *Ccnb1*, *c‐Mos*, and *Dazl*, upon stimulation of oocyte maturation (Guzeloglu‐Kayisli *et al*, 2012). In addition, late antral follicles in the ovaries of *Pabpc1l* ^−/−^ mice exhibited defective cumulus expansion and ovulation (Guzeloglu‐Kayisli *et al*, 2012; Yang *et al*, 2016). Similar to the observations in *Xenopus* and mice, human *PABPC1L* is the predominant PABP in oocytes (Guzeloglu‐Kayisli *et al*, 2008). To date, *PABPC1L* has not been found to be associated with any diseases in humans.

In this study, we identified compound heterozygous and homozygous pathogenic variants in *PABPC1L* (GRCh37, GenBank: NM_001124756.2) that are responsible for female infertility mainly characterized by oocyte maturation arrest. All five affected individuals carried biallelic variants, including missense, stop gain, or frameshift insertions, and all the five followed a Mendelian recessive inheritance pattern. We investigated the functional mechanism of the corresponding pathogenic variants in HeLa cells and *Pabpc1l* knock‐in (KI) mice. Our findings suggest a causal relationship between *PABPC1L* and female infertility and reveal important roles for PABPC1L in human oocyte maturation.

## Results

### Clinical characteristics of the affected individuals

**Figure 1:**
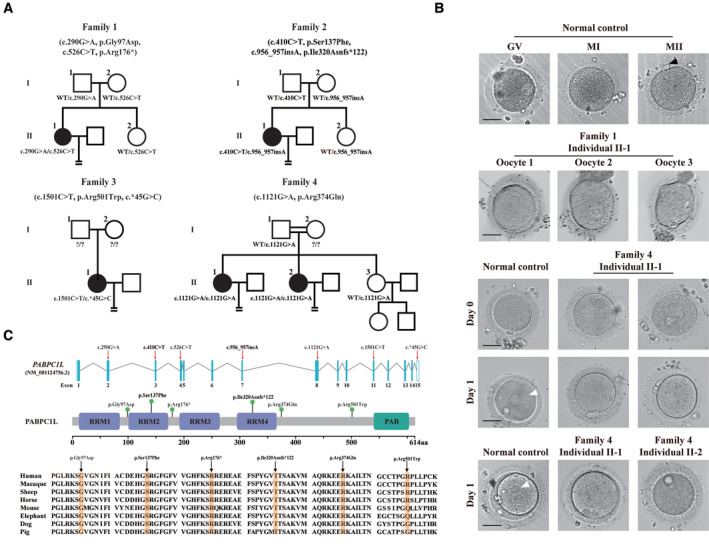
Identification of pathogenic variants in *PABPC1L* and the morphology of normal and affected individuals' oocytes Pedigrees of the four affected families. Squares indicate male members, circles indicate female members, black circles indicate the affected individuals, question marks indicate unavailable DNA samples, and equal signs (=) indicate infertility.The morphology of normal and affected individuals' oocytes or zygotes by light microscopy. The normal oocytes extruded PB1 (black arrowhead) and entered MII. Oocytes from individual II‐1 of family 1 were arrested at the GV or MI stage. The normal MII oocytes formed pronuclei (white arrowhead) on day 1 after ART. However, MII oocytes from individuals II‐1 and II‐2 of family 4 failed to fertilize and form pronuclei on day 1 after ART. Scale bar represents 40 μm.Locations and conservation of variants in *PABPC1L*. Red arrows and green balls indicate the variants identified in this study. PABPC1L contains four different RNA recognition motifs (RRMs) and a C‐terminal protein domain (PABP). Source data are available online for this figure.

To investigate the genetic factors responsible for oocyte maturation arrest, we recruited 1,394 infertile female individuals diagnosed with oocyte maturation arrest. In this study, all five affected individuals had been diagnosed with primary infertility of unknown cause for several years despite their having normal menstrual cycles. Their male partners had normal sperm counts and normal sperm morphologic features and motility. The pedigrees of the individuals are shown in Fig 1A. Clinical characteristic of affected individuals are shown in Table 1. The affected individual in family 1 underwent one failed IVF attempt. All the retrieved oocytes were arrested at the GV or MI stages (Fig 1B). The affected individual in family 2 underwent one failed IVF attempt and one failed ICSI attempt. In her IVF cycle, all retrieved oocytes were immature. In her ICSI cycle, 16 oocytes were obtained, of which 13 oocytes were immature. In addition, two mature MII oocytes could be fertilized, but the embryos arrested at the 1‐cell and 3‐cell stages, respectively. The affected individual in family 3 underwent one failed IVF attempt in which 11 oocytes were retrieved. Nine were arrested at the GV or MI stage. One mature MII oocyte was successfully fertilized, but this embryo was arrested at the 2‐cell stage. The affected individuals in family 4 underwent one failed IVF attempt and one failed ICSI attempt. In her IVF cycle, two MII oocytes were successfully fertilized but failed to cleave, and the remaining mature oocytes failed to fertilize. In her ICSI cycle, eight oocytes were arrested at the MI stage and the other two MII oocytes failed to fertilize (Fig 1B). Her sister underwent one failed IVF attempt in which 10 MII oocytes were retrieved but could not be fertilized (Fig 1B).

**Table 1:** Clinical characteristics of affected individuals and their retrieved oocytes.

Individual	Age (Years)	Duration of infertility (Years)	IVF/ICSI cycles	Total number of oocytes retrieved	GV oocytes	MI oocytes	MII oocytes	Oocytes with abnormal morphology	Fertilized oocytes	Cleaved embryos
II‐1 in Family 1	25	4	IVF	7	5	2	0	0	0	0
II‐1 in Family 2	33	7	IVF	21	5	8	0	8	0	0
II‐1 in Family 2	33	7	ICSI	16	3	4	3	6	2	1
II‐1 in Family 3	29	4	IVF	11	7	2	2	0	1	1
II‐1 in Family 4	33	6	IVF	11	0	1	10	0	2	0
II‐1 in Family 4	33	6	ICSI	11	0	8	2	1	0	0
II‐2 in Family 4	29	3	IVF	15	0	4	10	1	0	0

Abbreviations are as follows: GV, germinal vesicle; MI, metaphase I; and MII, metaphase II.

### Identification of bi‐allelic pathogenic variants in *PABPC1L*

**Figure EV1:**
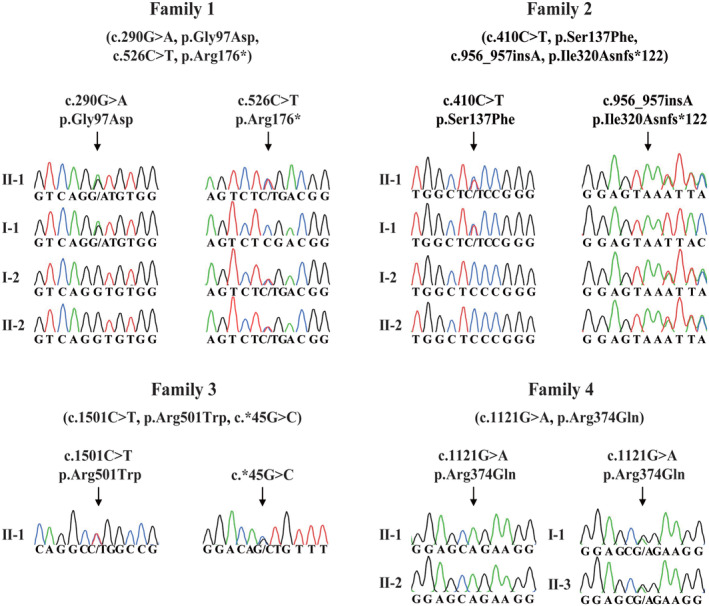
Sanger sequencing confirmation of *PABPC1L* variants in four families *PABPC1L* variants observed in the four families were confirmed using Sanger sequencing. Arrows indicate the mutation sites.Source data are available online for this figure.

**Figure EV2:**
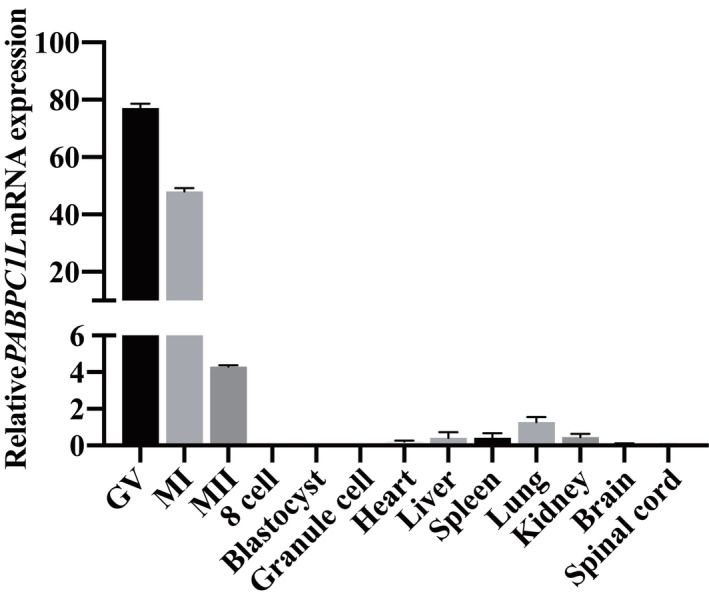
Relative expression level of *PABPC1L* mRNA in different stages of human oocytes, embryos and several somatic tissues The relative expression of *PABPC1L* mRNA in different stages of human oocytes, early embryos, and several somatic tissues was measured by qRT–PCR and normalized to the expression of *GAPDH* mRNA (control). *n* = 3 biological replicates. Error bars denote the SD.Source data are available online for this figure.

We performed whole‐exome sequencing and bioinformatics analyses in accordance with our established protocol (see Material and Methods) to identify potential genetic causes of the oocyte maturation arrest observed in our patients. We first identified three infertile individuals from three independent families (families 1–3) harboring bi‐allelic variants in *PABPC1L* (GenBank: NM_001124756.2) (Fig 1A). No homozygous or compound heterozygous variants in *PABPC1L* were found in our control database. The affected individual in family 1 carried biallelic variants c.290G>A (p.Gly97Asp) and c.526C>T (p.Arg176*). The affected individual in family 2 carried the compound heterozygous variants c.410C>T (p.Ser137Phe) and c.956_957insA (p.Ile320Asnfs*122), whereas the affected individual in family 3 carried the variants c.1501C>T (p.Arg501Trp) and 3′UTR variant c.*45G>C. These results implied the genetic contribution of *PABPC1L* to oocyte maturation arrest and female infertility. We then performed mutational screening of *PABPC1L* in a cohort of 504 infertile individuals with abnormalities in fertilization and early embryonic development. We identified two affected sisters from family 4 who carried the same homozygous missense variant c.1121G>A (p.Arg374Gln). All identified variants were confirmed using Sanger sequencing (Fig EV1). The inheritance pattern in family 3 was unknown due to the unavailability of DNA samples from her parents, whereas the other three families followed a recessive inheritance pattern (Fig 1A). Specific information about the variants, including the genomic position, predicted damaging effect, and frequency, is provided in Table 2. *PABPC1L* contains 15 exons encoding a 614 amino acid protein, and the locations of the variants in the gene and protein structure are shown in Fig 1C. Most of the corresponding residues affected by the variants are conserved among different species, whereas the variant c.1501C>T (p.Arg501Trp) is only conserved in some species (Fig 1C). We measured the expression of *PABPC1L* mRNA in control samples and found that *PABPC1L* was highly expressed in human immature GV and MI oocytes, but was poorly expressed in mature MII oocyte, early embryos, and other somatic tissues (Fig EV2), suggesting an important role for *PABPC1L* in human oocyte maturation.

**Table 2:** *PABPC1L* pathogenic variants observed in the four families.

Probands in families	Genomic position on chr20 (bp)	cDNA change	Protein change	Mutation type	SIFT^a^	PPH2^a^	MutTas^a^	gnomAD^b^
Family 1	43541397	c.290G>A	p.Gly97Asp	missense	D	P	D	NA
	43547569	c.526C>T	p.Arg176*	stop gain	NA	NA	D	4.2 × 10^−6^
Family 2	43545419	c.410C>T	p.Ser137Phe	missense	D	P	D	4.0 × 10^−6^
	43552881	c.956_957insA	p.Ile320Asnfs*122	frameshift insertion	NA	NA	D	NA
Family 3	43564088	c.1501C>T	p.Arg501Trp	missense	D	P	P	8.0 × 10^−6^
	43567805	c.*45G>C	–	missense	NA	NA	P	7.2 × 10^−4^
Family 4	43559249	c.1121G>A	p.Arg374Gln	missense	D	P	D	1.2 × 10^−5^

*PABPC1L*—poly(A)‐binding protein cytoplasmic 1 like.

^a^ Mutation assessment by SIFT, PolyPhen‐2 (PPH2), and MutationTaster (MutTas). D, damaging; P, probably damaging; and NA, not available.

^b^ Allele frequency of corresponding mutations in the gnomAD database. NA, not available.

### Pathogenic variants in *PABPC1L* impaired protein expression and localization in HeLa cells

**Figure 2:**
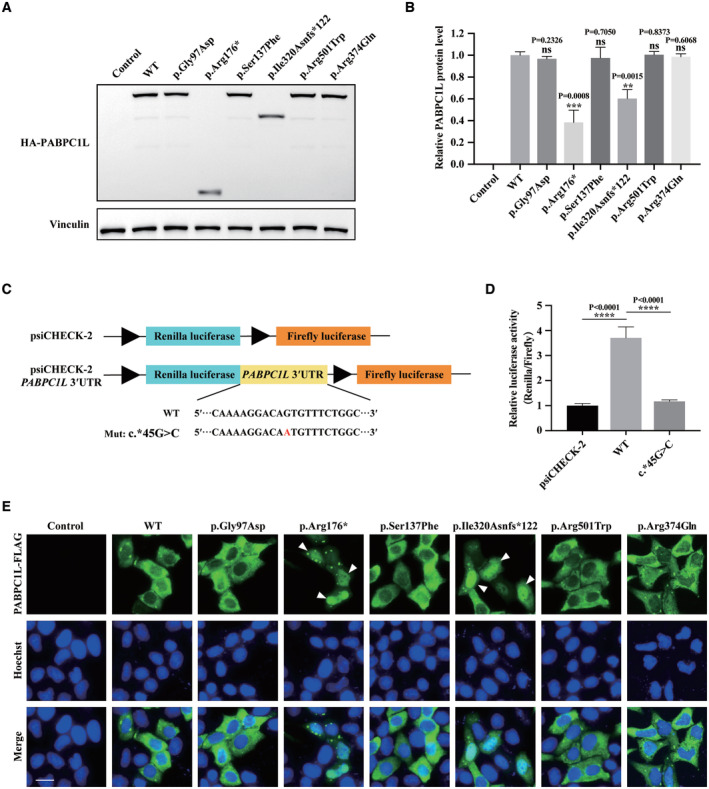
Effects of *PABPC1L* pathogenic variants on protein abundance and localization in HeLa cells The effects of the pathogenic variants on *PABPC1L* expression by immunoblot in HeLa cells. Empty vector was used as the control. Vinculin was used as the loading control.Quantitation of the Control, WT, and mutant PABPC1L protein levels shown in Fig 3A. *n* = 3 biological replicates. The statistics are analyzed by unpaired two‐tailed Student's *t*‐test. Data are shown as the mean and SD. **P < 0.005, ***P < 0.001.Schematic representation of dual luciferase reporter plasmid psiCHECK‐2 vectors containing the WT and mutant *PABPC1L* 3′UTR inserted downstream of the Renilla luciferase gene. The mutant nucleotide is marked in red.HeLa cells were transfected with luciferase reporter constructs. Empty vector was used as the control. Relative Renilla luciferase activity was determined and normalized to Firefly luciferase activity. *n* = 4 biological replicates. The statistics are analyzed by unpaired two‐tailed Student's *t*‐test. Data are shown as the mean and SD. ****P < 0.0001.HeLa cells were transfected with FLAG‐tagged full‐length WT or mutant *PABPC1L* expression plasmids and stained with an anti‐FLAG M2‐FITC antibody (green). Nuclei were labeled with Hoechst (blue). White arrowheads indicate that the p.Arg176* and p.Ile320Asnfs*122 mutant proteins were mostly mislocalized in the nucleus compared to the typical cytoplasmic localization for WT protein. Scale bar represents 20 μm. Source data are available online for this figure.

To evaluate the functional effects of the identified pathogenic variants *in vitro*, we first performed immunoblot analysis in HeLa cells transfected with HA‐labeled WT or mutant *PABPC1L* expression plasmids. Compared with WT, the c.526C>T (p.Arg176*) and c.956_957insA (p.Ile320Asnfs*122) variants resulted in truncated proteins and showed significantly reduced PABPC1L protein levels. The missense variants c.290G>A (p.Gly97Asp), c.410C>T (p.Ser137Phe), c.1501C>T (p.Arg501Trp), and c.1121G>A (p.Arg374Gln) did not lead to changes in protein levels (Fig 2A and B). Furthermore, to determine whether the 3′UTR variant c.*45G>C affects gene expression, we cloned WT and mutant 3′UTR segments into a dual luciferase reporter plasmid psiCHECK‐2 vector (Fig 2C). The luciferase assay in HeLa cells indicated that the WT 3′UTR significantly increased the reporter gene expression, whereas the mutant 3′UTR failed to up‐regulate the reporter gene expression compared to the empty vector (Fig 2D). This result suggests that the 3′UTR variant c.*45G>C may affect the expression of PABPC1L.

It has been reported that PABPC1L is mainly localized in the cytoplasm in mouse oocytes (Uysal & Ozturk, 2019). The subcellular localization of proteins is important for their function, so to further investigate whether the identified variants affect the function of PABPC1L, immunofluorescence was performed using a commercial anti‐FLAG antibody in HeLa cells transfected with FLAG‐tagged WT or mutant *PABPC1L* expression plasmids. As shown in Fig 2E, the WT PABPC1L was mainly localized in the cytoplasm, while the variants c.526C>T (p.Arg176*) and c.956_957insA (p.Ile320Asnfs*122) resulted in mislocalization of most protein in the nucleus. The missense variants c.290G>A (p.Gly97Asp), c.410C>T (p.Ser137Phe), c.1501C>T (p.Arg501Trp), and c.1121G>A (p.Arg374Gln) had no effect on protein cytoplasmic localization. These results demonstrate that the identified pathogenic variants c.526C>T (p.Arg176*) and c.956_957insA (p.Ile320Asnfs*122) affect both the protein abundance and localization of PABPCL, while the 3′UTR variant c.*45G>C affects the protein expression of the reporter gene in HeLa cells.

### Pathogenic variants in *PABPC1L* disrupted its mRNA translational activation and RNA‐binding ability in HeLa cells

**Figure 3:**
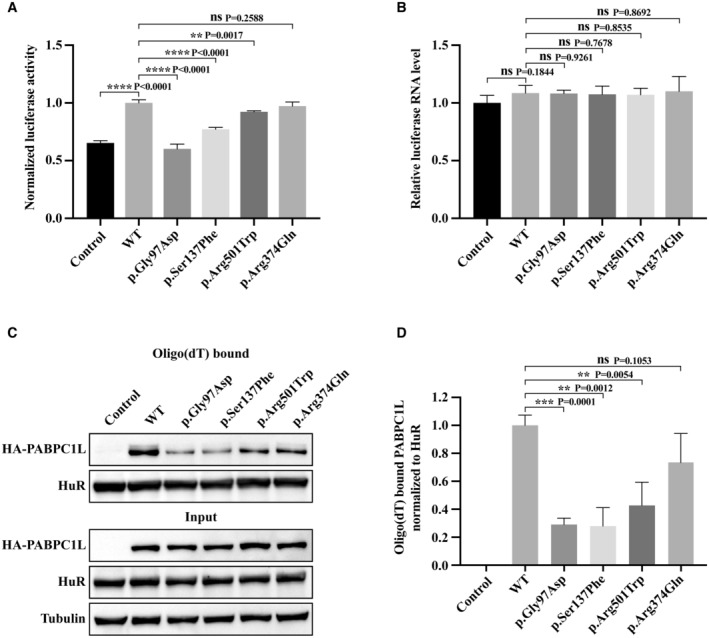
Effects of *PABPC1L* pathogenic variants on its mRNA translational activation and RNA‐binding ability in HeLa cells HeLa cells were transfected with FLB reporter (Firefly luciferase fused to β‐globin) together with WT or mutant *PABPC1L* expression plasmids, and cell extracts were assayed for Firefly luciferase activity. Empty vector was used as the control. *n* = 4 biological replicates. The statistics are analyzed by unpaired two‐tailed Student's *t*‐test. Data are shown as the mean and SD. **P < 0.01, ****P < 0.0001.FLB mRNA levels were determined from the same cell extracts and normalized to the *GAPDH* mRNA level. *n* = 3 biological replicates. The statistics are analyzed by unpaired two‐tailed Student's *t*‐test. Data are shown as mean and SD. ns, not significant.Representative immunoblots for WT and mutant PABPC1L bound to RNA in HeLa cells. HuR was used as the internal control, and Tubulin was used as the loading control.Quantitation of the oligo‐dT–bound WT or mutant PABPC1L signal normalized to the HuR signal. *n* = 4 biological replicates. The statistics are analyzed by unpaired two‐tailed Student's *t*‐test. Data are shown as the mean and SD. **P < 0.01, ***P < 0.001, ns, not significant. Source data are available online for this figure.

PABPC1L is an RNA‐binding protein that promotes the translational activation of maternal RNA in *Xenopus* and mouse oocytes, which is required for oocyte maturation (Wilkie *et al*, 2005; Guzeloglu‐Kayisli *et al*, 2012). Because the missense variants c.290G>A (p.Gly97Asp), c.410C>T (p.Ser137Phe), c.1501C>T (p.Arg501Trp), and c.1121G>A (p.Arg374Gln) had no effect on protein level or localization, we speculated that these variants might influence the protein's ability to promote mRNA translational activation. Therefore, we compared the translational activation capacity of WT and mutant PABPC1L by transfecting a luciferase reporter together with WT or mutant *PABPC1L* expression plasmids in HeLa cells. All of the variants except c.1121G>A (p.Arg374Gln) resulted in significantly lower luciferase activity than the WT group (Fig 3A), while luciferase mRNA levels were not significantly different between the groups (Fig 3B). These results indicate that the missense variants c.290G>A (p.Gly97Asp), c.410C>T (p.Ser137Phe), and c.1501C>T (p.Arg501Trp) led to a significant decrease in mRNA translational activation in HeLa cells.

PABPC1L promotes the translational activation of mRNAs by interacting with the poly(A) tail of mRNA (Voeltz *et al*, 2001; Cao & Richter, 2002). To determine whether the missense variants affect PABPC1L's RNA‐binding ability, we performed RNA pull‐down experiments in HeLa cells. The results showed that the variants c.290G>A (p.Gly97Asp), c.410C>T (p.Ser137Phe), and c.1501C>T (p.Arg501Trp) had dramatically reduced RNA‐binding ability compared to WT (Fig 3C and D), indicating that these variants resulted in reduced mRNA translation activation by affecting the binding of PABPC1L to RNA. The variant c.1121G>A (p.Arg374Gln) also had a slight effect on RNA binding, but the difference was not significant (Fig 3C and D). The above *in vitro* data demonstrate that the identified pathogenic variants in *PABPC1L* have disruptive effects on protein function.

### *Pabpc1l* KI female mice exhibit early embryonic arrest and infertility

**Figure 4:**
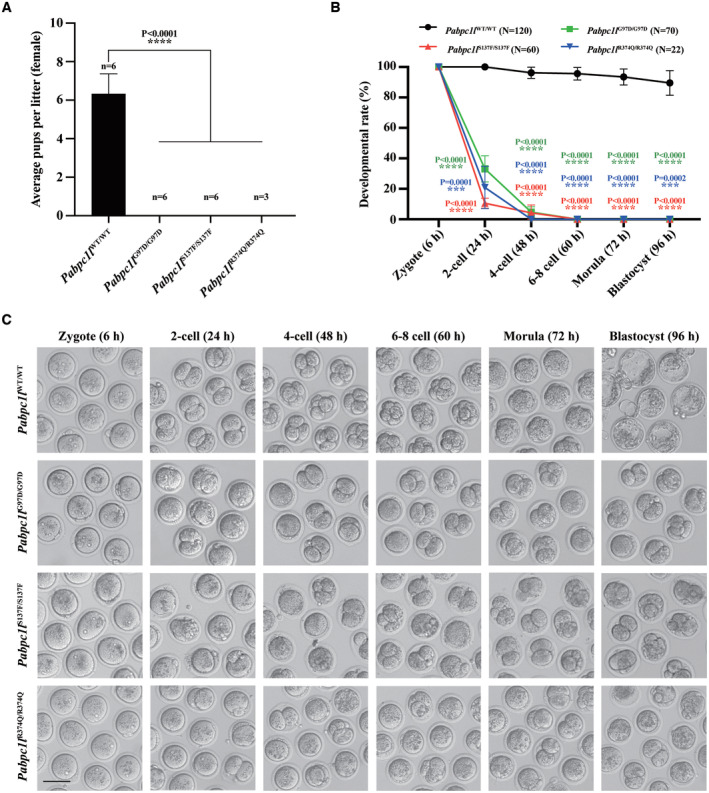
*Pabpc1l* KI female mice exhibit abnormal early embryonic development and infertility The reproductive ability of female WT and *Pabpc1l* KI mice. *n* = 6 for *Pabpc1l* ^WT/WT^, *Pabpc1l* ^G97D/G97D^, and *Pabpc1l* ^S137F/S137F^ mice. *n* = 3 for *Pabpc1l* ^R374Q/R374Q^ mice. The statistics are analyzed by unpaired two‐tailed Student's *t*‐test. Data are shown as mean and SD. ****P < 0.0001.Quantification of early embryonic development at different times after IVF of superovulated oocytes from WT and *Pabpc1l* KI mice. The number of analyzed embryos is indicated (*N*). *n* = 4 biological replicates for *Pabpc1l* ^WT/WT^, *Pabpc1l* ^G97D/G97D^, and *Pabpc1l* ^S137F/S137F^ mice. *n* = 2 biological replicates for *Pabpc1l* ^R374Q/R374Q^ mice. The statistics are analyzed by unpaired two‐tailed Student's *t*‐test. Data are shown as the mean and SD. ***P < 0.001, ****P < 0.0001.Representative images of early embryonic development at different times after IVF of superovulated oocytes from WT and *Pabpc1l* KI mice. Scale bar represents 100 μm. Source data are available online for this figure.

**Figure EV3:**
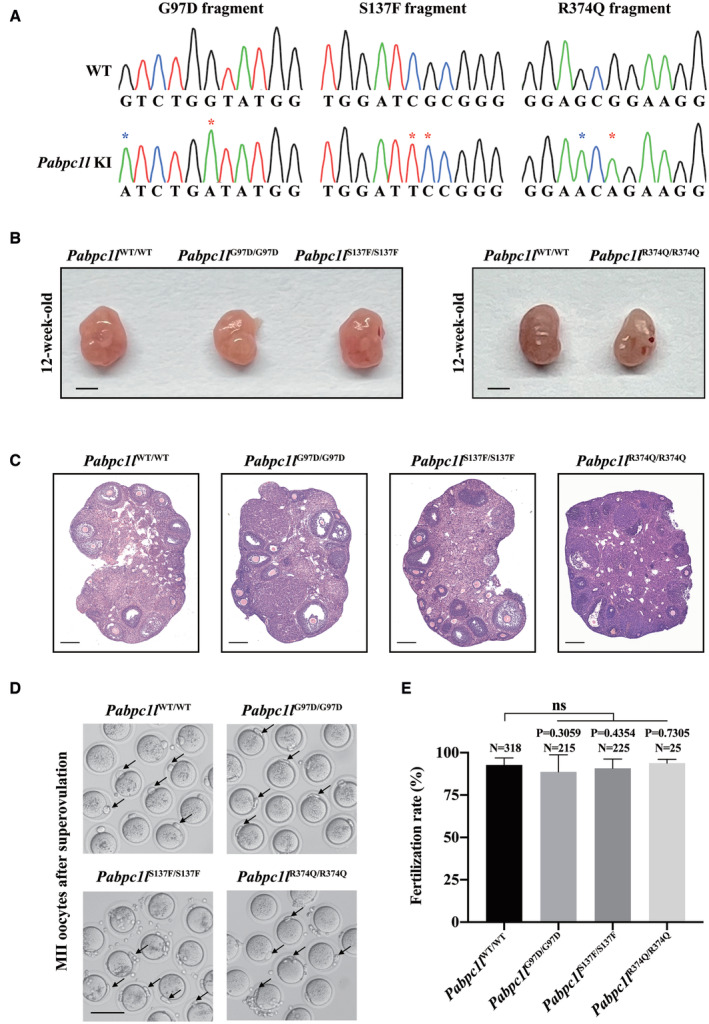
Genotyping and phenotype analyses in homozygous *Pabpc1l* KI mice Sanger sequencing showing the homozygous mutations in *Pabpc1l* KI mice. Red asterisks indicate the mutation sites, and the blue asterisk indicates the synonymous mutation site.Ovary morphology of 12‐week‐old WT and *Pabpc1l* KI mice. Scale bar represents 1 mm.Histological sections of ovaries from 12‐week‐old WT and *Pabpc1l* KI mice were stained with hematoxylin and eosin. Scale bars represent 200 μm.Representative images of superovulated oocytes of WT and *Pabpc1l* KI mice. The black arrows indicate PB1. Scale bar represents 100 μm.Quantitative analysis of the fertilization rate of WT and *Pabpc1l* KI mice. The number of analyzed embryos is indicated (*N*). *n* = 8 biological replicates for *Pabpc1l* ^WT/WT^, *Pabpc1l* ^G97D/G97D^, and *Pabpc1l* ^S137F/S137F^ mice. *n* = 2 biological replicates for *Pabpc1l* ^R374Q/R374Q^ mice. The statistics are analyzed by unpaired two‐tailed Student's *t*‐test. Data are shown as mean and SD. ns, not significant.

To further explore the pathogenic role of *PABPC1L* variants *in vivo*, we utilized the CRISPR‐Cas9 system to generate *Pabpc1l* G97D, S137F, and R374Q KI mice corresponding to the pathogenic variants c.290G>A (p.Gly97Asp), c.410C>T (p.Ser137Phe), and c.1121G>A (p.Arg374Gln) identified in families 1, 2, and 4 respectively. We performed PCR and Sanger sequencing to confirm the mutated allele in the three KI mice (Fig EV3A). All three *Pabpc1l* KI mice were viable, but females were infertile (Fig 4A). To gain insights into the etiology of infertility in females, we first assessed the ovarian histology of unstimulated mature WT and *Pabpc1l* KI mice, and no differences were observed between them (Fig EV3B and C). Next, we performed phenotypic analysis of *Pabpc1l* KI females in terms of oocyte maturation, fertilization, and early embryonic development. Different from the phenotype observed in affected individuals with these three variants, *Pabpc1l* KI females ovulated mature MII oocytes after superovulation treatment (Fig EV3D). Furthermore, the fertilization rate of *Pabpc1l* KI oocytes was normal (Fig EV3E), and they formed pronuclei at 6 h after IVF (Fig 4C). However, in contrast to the WT zygotes, the cleavage of *Pabpc1l* KI zygotes was delayed, and most KI zygotes could cleavage in the subsequent *in vitro* culture, but all embryos eventually arrested at an early stage and failed to develop into blastocysts (Fig 4B and C). These results further indicated that the *PABPC1L* variants are indeed pathogenic for female fertility in the affected individuals and that there are species‐specific differences in the effects of mutant *PABPC1L* in humans and mice.

### Abnormal activation of the Mos‐MAPK pathway in zygotes causes early embryonic arrest in *Pabpc1l* KI mice

**Figure 5:**
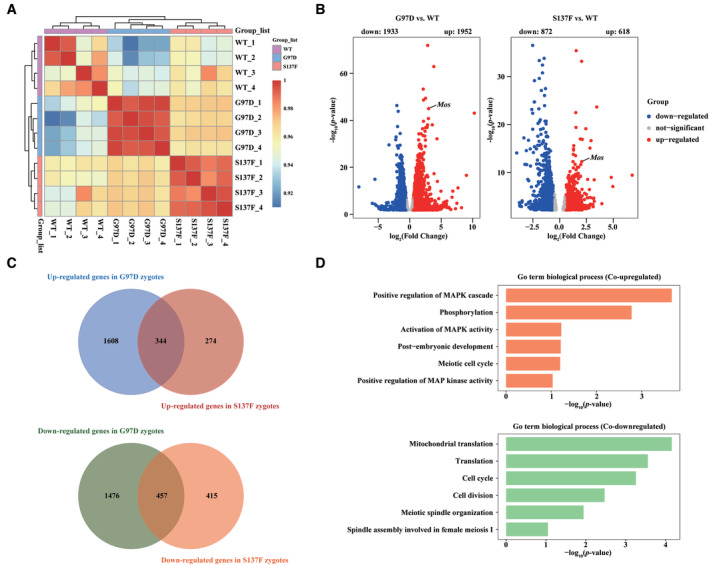
RNA‐seq analysis of the transcriptome of WT and *Pabpc1l* KI zygotes Heatmap of the Spearman correlation coefficients in the RNA‐seq data obtained from WT, G97D, and S137F zygotes.Volcano plot of RNA‐seq data obtained from WT, G97D, and S137F zygotes. “Up” and “Down” are the upregulated and downregulated transcripts in KI zygotes compared with WT zygotes. The genes with upregulation and downregulation (abs[Fold Change] > 1.5, FDR <0.05) were labeled in red and blue, respectively.Venn diagram showing the number of co‐upregulated and co‐downregulated genes for G97D vs. WT and S137F vs. WT.GO analysis of co‐upregulated and co‐downregulated genes in G97D vs. WT and S137F vs. WT.

To explore the mechanism of early embryonic arrest in *Pabpc1l* KI mice, we performed RNA‐seq using WT and *Pabpc1l* KI zygotes. Gene expression levels were assessed as FPKM, and all replicates showed high correlations (Fig 5A). Compared to WT zygotes, 1,933 transcripts were downregulated and 1,952 transcripts were upregulated in G97D zygotes, while 872 transcripts were downregulated and 618 transcripts were upregulated in S137F zygotes (Fig 5B). Furthermore, Venn diagrams showed that there were 344 co‐upregulated transcripts and 457 co‐downregulated transcripts in the two groups (G97D vs. WT and S137F vs. WT; Fig 5C). GO analysis revealed that the co‐upregulated genes were mainly involved in positive regulation of mitogen‐activated protein kinase (MAPK) cascade pathways, phosphorylation, embryonic development, etc, while the co‐downregulated genes were involved in translation, cell cycle, meiotic spindle organization, etc. (Fig 5D). To further investigate the molecular mechanism, we first analyzed the differentially expressed genes in G97D zygotes and mainly focused on oocyte‐specific genes. We found that *Mos*, an upstream activator of MAPK cascade (Dupre *et al*, 2011), was the only oocyte‐specific gene among the top 30 differentially expressed genes (Dataset EV1; Fig 5B). Then we checked the expression of *Mos* in S137F zygotes and observed that it was also up‐regulated (Dataset EV1; Fig 5B). The up‐regulation of *Mos* in KI zygotes is consistent with the activation of MAPK cascade revealed in GO analysis. The above RNA‐seq analysis results strongly suggest that Mos‐MAPK pathway is abnormally activated in KI zygote. In addition, studies have indicated that abnormal activation of Mos‐MAPK pathway in embryo led to cleavage arrest (Sagata *et al*, 1989; Haccard *et al*, 1993; Verlhac *et al*, 2000), which is similar to the phenotype of early embryonic arrest in KI mice. Therefore, we supposed that the abnormal up‐regulation of *Mos* expression and activation of the Mos‐MAPK pathway in KI zygotes might be the potential mechanism of early embryonic arrest in *Pabpc1l* KI mice.

**Figure 6:**
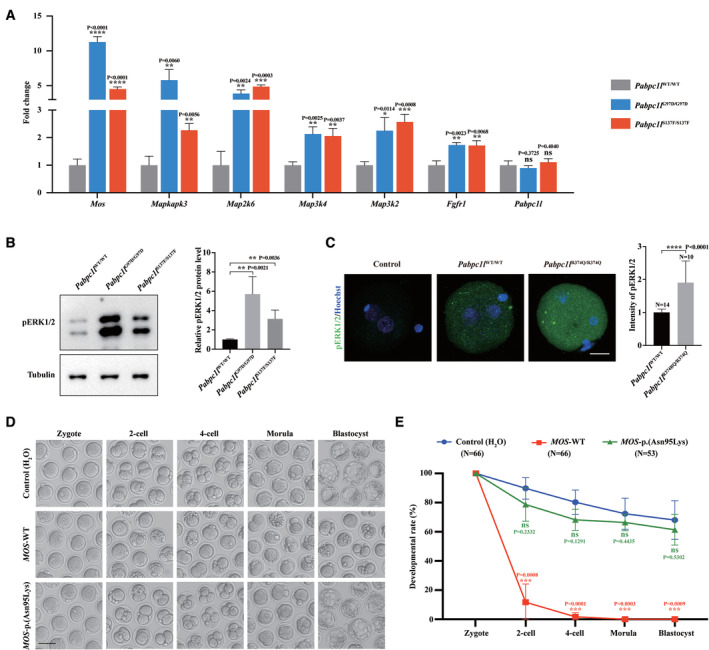
*Pabpc1l* pathogenic variants cause abnormal activation of the Mos‐MAPK pathway in zygotes and lead to early embryonic arrest in KI mice qRT–PCR results verified the expression of upregulated Mos‐MAPK pathway‐related genes and *Pabpc1l* in the RNA‐seq data. *n* = 3 biological replicates. The statistics are analyzed by unpaired two‐tailed Student's *t*‐test. Data are shown as the mean and SD. *P < 0.01, **P < 0.005, ***P < 0.001, ****P < 0.0001, ns, not significant.Immunoblot analysis of pERK1/2 in WT, G97D, and S137F zygotes. Total protein from 50 zygotes was loaded in each lane. Tubulin was used as the loading control. *n* = 4 biological replicates. The statistics are analyzed by unpaired two‐tailed Student's *t*‐test. Data are shown as the mean and SD. **P < 0.005.Immunofluorescence analysis of pERK1/2 (green) in WT and R374Q zygotes. Hoechst (blue) was co‐stained to represent pronucleus. Scale bar represents 20 μm. The number of analyzed embryos is indicated (*N*). *n* = 2 biological replicates. The statistics are analyzed by unpaired two‐tailed Student's *t*‐test. Data are shown as the mean and SD. ****P < 0.0001.Representative images of early embryonic development at different times after WT zygotes were injected with H_2_O (Control), *MOS‐*WT, or *MOS*‐p.(Asn95Lys) cRNA. Scale bar represents 100 μm.Quantification of early embryonic development at different times after WT zygotes were injected with H_2_O (Control), *MOS*‐WT, or *MOS*‐p.(Asn95Lys) cRNA. The number of analyzed embryos is indicated (*N*). *n* = 3 biological replicates. The statistics are analyzed by unpaired two‐tailed Student's *t*‐test. Data are shown as the mean and SD. ***P < 0.001, ns, not significant. Source data are available online for this figure.

To determine whether Mos‐MAPK pathway is indeed abnormally activated in KI zygote, we first performed qRT‐PCR to confirm the RNA‐seq data. qRT‐PCR results showed that the expression levels of *Mos* and MAPK pathway‐related gene in G97D and S137F zygotes were indeed higher than those in WT zygotes, while the expression levels of *Pabpc1l* showed no differences between groups (Fig 6A). Phosphorylated ERK1/2 (pERK1/2) is a well‐known marker of MAPK pathway activation (Cao *et al*, 2021). To further confirm whether the MAPK pathway is activated in *Pabpc1l* KI zygotes, we collected the zygotes to examine the phosphorylation level of ERK1/2. As shown in Fig 6B and C, the pERK1/2 levels were significantly elevated in G97D, S137F, and R374Q zygotes compared with WT zygotes. These results demonstrate that the Mos‐MAPK pathway is indeed abnormally activated in *Pabpc1l* KI zygotes.

The Mos‐MAPK pathway plays a crucial role in cytostatic factor‐induced oocyte MII arrest (Haccard *et al*, 1993; Colledge *et al*, 1994; Kosako *et al*, 1994). Previous studies in *Xenopus* and mice showed that injection of *Mos* mRNA into the blastomeres of two‐cell embryos could induce the abnormal activation of Mos‐MAPK pathway in blastomeres and eventually resulted in cleavage arrest. (Sagata *et al*, 1989; Verlhac *et al*, 2000). Thus, we microinjected zygotes with human *MOS* cRNA to activate the Mos‐MAPK pathway and observed the resulting embryonic development. Compared with the control group, injection of *MOS*‐WT cRNA caused early embryonic arrest (Fig 6D and E), mimicking the phenotype of *Pabpc1l* KI mice. However, injection of *MOS*‐p.(Asn95Lys) cRNA (a previously reported loss‐of‐function variant) (Zeng *et al*, 2022) did not result in the phenotype (Fig 6D and E). Together, these results indicate that *PABPC1L* pathogenic variants lead to increased *Mos* expression and abnormal activation of Mos‐MAPK pathway in zygotes, ultimately resulting in early embryonic arrest and female infertility in *Pabpc1l* KI mice.

## Discussion

In this study, we identified different compound heterozygous and homozygous pathogenic variants in *PABPC1L* that are mainly responsible for human oocyte maturation arrest. *In vitro* studies showed that the truncating variants c.526C>T (p.Arg176*) and c.956_957insA (p.Ile320Asnfs*122) affected the expression pattern and localization of PABPC1L, while the missense variants c.290G>A (p.Gly97Asp), c.410C>T (p.Ser137Phe), and c.1501C>T (p.Arg501Trp) reduced its translation activation and RNA‐binding ability. Moreover, the 3′UTR variant c.*45G > C affected the protein expression of the luciferase reporter gene. Finally, we constructed three strains of *Pabpc1l* KI mice corresponding to patient‐derived variants and found that female mice were infertile. The Mos‐MAPK pathway was abnormally activated in the zygotes of KI mice, which may provide an explanation the infertile phenotype. These results demonstrate the destructive effects and pathogenicity of the *PABPC1L* variants identified in affected individuals both *in vitro* and *in vivo*.

Cytoplasmic PABPs (poly(A)‐binding proteins) are a family of multifunctional RNA‐binding proteins, including PABPC1, PABPC3, PABPC4, PABPC4L, PABPC5, and PABPC1L, that mediate mRNA metabolism at different stages of gametogenesis and early embryogenesis (Zhao & Fan, 2021). PABPC1L was initially discovered in 2001 as a novel ARE (AU‐rich element) binding protein in *Xenopus* oocyte extracts that regulates mRNA deadenylation, and it is the predominant PABP in *Xenopus* oocytes and early embryos (Voeltz *et al*, 2001). In the last two decades, the physiological function of the PABPC1L‐mediated translational activation of maternal mRNA in oocyte and early embryo has been well characterized in studies in *Xenopus* and mice. The amino acid sequence of PABPC1L is highly conserved in *Xenopus* and mammals, and *PBAPC1L* is also the predominant PABP in human oocytes and early embryos (Guzeloglu‐Kayisli *et al*, 2008). However, the physiological function of human PABPC1L is unknown. In this study, we showed that *PABPC1L* was highly expressed in human immature oocytes (Fig EV2) and that WT human PABPC1L could significantly stimulate the mRNA translation of reporter gene *in vitro* (Fig 3A). On the other hand, patient‐derived variants (c.290G>A (p.Gly97Asp), c.410C>T (p.Ser137Phe), and c.1501C>T (p.Arg501Trp)) disrupted the translational activation of PABPC1L (Fig 3A) and resulted in female infertility characterized by oocyte maturation arrest (Fig 1B and Table 1). Therefore, these results suggest that PABPC1L plays an important role in mRNA translational activation during human oocyte maturation and that it is essential for female fertility.

Previous studies have found that patients with different variants in the same oocyte maturation arrest‐related genes, including *TUBB8*, *PATL2* and *TRIP13*, exhibit various phenotypes (Chen *et al*, 2019; Wu *et al*, 2019; Zhang *et al*, 2020). This may be due to different variants having different effects on protein function. In this study, all retrieved oocytes of the affected individual from family 1 were arrested at GV or MI stages (Fig 1B and Table 1). However, a few retrieved oocytes of the affected individuals in families 2 and 3 were able to mature and be fertilized, but they arrested at the 1‐cell to 3‐cell stages prior to ZGA (Table 1). PABPC1L plays a key role in the polyadenylation‐dependent translational activation of mRNAs, which is essential for oocyte maturation (Wakiyama *et al*, 2000; Guzeloglu‐Kayisli *et al*, 2012). Our functional experiment demonstrated that the pathogenic variant c.290G>A (p.Gly97Asp) from family 1 was significantly more disruptive to the translational activation ability of PABPC1L than variants c.410C>T (p.Ser137Phe) and c.1501C>T (p.Arg501Trp) from families 2 and 3 (Fig 3A). This suggests that the severity of the phenotype in affected individuals with *PABPC1L* variants depends on the degree of impairment of PABPC1L function resulting from the different variants. Furthermore, all embryos obtained from the affected individuals in families 2 and 3 were arrested at an early stage, suggesting that PABPC1L also play an important role in early embryonic development prior to ZGA.

For individuals II‐1 and II‐2 in consanguineous family 4, some oocytes obtained from ART were also immature and arrested at the MI stage. However, unlike the affected individuals in other families, most of their oocytes could extrude the PB1 to develop into mature MII oocytes but showed subsequent fertilization failure (Table 1). The two individuals carried the same homozygous variant c.1121G>A (p.Arg374Gln) in *PABPC1L* and showed the same infertile phenotype, while individual II‐3 carried the same heterozygous variant c.1121G>A (p.Arg374Gln) and had normal fertility. This result suggests that the c.1121G>A (p.Arg374Gln) variant may cause the phenotype of fertilization failure following a recessive inheritance pattern. The unique fertilization failure phenotype might be related to the observation that the c.1121G>A (p.Arg374Gln) variant had a slight effect on the RNA‐binding ability of PABPC1L (Fig 3B) but had no effect on mRNA translational activation (Fig 3A). To further confirm the pathogenicity of the variant c.1121G>A (p.Arg374Gln), we constructed *Pabpc1l* R374Q KI mice corresponding to this variant. Phenotypic evaluation found that *Pabpc1l* ^R374Q/R374Q^ female mice were infertile characterized by early embryonic arrest (Fig 4A–C), which was consistent with the other two KI mice. In addition, immunofluorescence analysis showed that the level of pERK1/2 in the R374Q KI zygotes was significantly increased (Fig 6C), indicating that the Mos‐MAPK pathway was also abnormally activated. These results fully demonstrate that the variant c.1121G>A (p.Arg374Gln) identified in family 4 has disruptive effects on protein function and ultimately leads to female infertility. We inferred that the biochemical mechanism of Arg374Gln *in vitro* may be different from other variants, but all variants have the similar pathological mechanism *in vivo*.

*Pabpc1l* knock‐out (KO) female mice were previously shown to be infertile due to impaired oocyte maturation (Guzeloglu‐Kayisli *et al*, 2012). In this study, we constructed three strains of *Pabpc1l* KI mouse models – *Pabpc1l* ^G97D/G97D^ *Pabpc1l* ^S137F/S137F^, and *Pabpc1l* ^R374Q/R374Q^ – and unlike the phenotype of KO mice, the retrieved oocytes from KI mice matured and could be fertilized normally (Fig EV3D and E), but the embryos developed abnormally and most embryos were arrested at the 2‐cell stage prior to ZGA (Fig 4B and C). One possible explanation for this is that the mutant PABPC1L still has some function during oocyte maturation, but this activity is not sufficient for further early embryonic development after fertilization. Our Immunoblot and immunofluorescence analysis showed that the missense variants c.290G>A (p.Gly97Asp), c.410C>T (p.Ser137Phe), and c.1121G>A (p.Arg374Gln) had no effect on protein levels and cytoplasmic localization of PABPC1L (Fig 2A and E). In addition, RNA pull‐down assay indicated that the pathogenic variants c.290G>A (p.Gly97Asp) and c.410C>T (p.Ser137Phe) resulted in a significant decrease in the RNA‐binding ability of PABPC1L compared to WT; however, there was still a small amount of mutant PABPC1L bound to RNA (Fig 3C and D). This further suggests that the phenotypic severity of individuals with *PABPC1L* variants correlates with the relative loss of function of the variants.

MOS is a serine/threonine kinase that activates the MAPK cascade through direct phosphorylation of the MAP kinase activator MEK (Posada *et al*, 1993). The Mos‐MAPK pathway is commonly activated during vertebrate oocyte maturation, but its function seems to be different among species. In *Xenopus* oocytes, the activation of the Mos‐MAPK pathway is necessary for triggering GVBD and for cytostatic factor‐induced MII arrest (Sagata *et al*, 1989; Haccard *et al*, 1993; Posada *et al*, 1993). However, *Mos* deletion in mouse oocytes has shown that the Mos‐MAPK pathway is not required for GVBD but is important for MII arrest and female fertility (Colledge *et al*, 1994; Choi *et al*, 1996; Hashimoto, 1996). In this study, we demonstrated that *PABPC1L* pathogenic variants lead to increased *Mos* expression and abnormal activation of the Mos‐MAPK pathway in mouse zygotes. Considering that the affected individuals with variants in *PABPC1L* showed either oocyte maturation arrest or fertilization failure, we believe that dysfunction of PABPC1L may lead to abnormal MOS‐MAPK pathway activity and ultimately affect human oocyte maturation and fertilization processes.

This study has some limitations. First, this study lacks experimental analysis of oocytes or embryos from affected individuals with *PABPC1L* variants, because we have not obtained valuable clinical samples from these patients. Second, although we have confirmed the pathogenicity of the *PABPC1L* variants and provided the exploration of the mechanism leading to phenotype, we have not found a possible treatment to overcome infertility. Previous studies have found that microinjection of *Pabpc1l* mRNA into *Pabpc1l* ^−/−^ preantral follicle‐enclosed oocytes rather than denuded GV oocytes could rescue oocyte maturation (Guzeloglu‐Kayisli *et al*, 2012; Lowther & Mehlmann, 2015). This strategy might provide a possible treatment option to overcome female infertility caused by PABPC1L dysfunction, but additional experiments should be pursed for evaluating effectiveness to embryonic development.

In this study, we identified five individuals with bi‐allelic variants in *PABPC1L*, which accounts for 0.26% of our cohort of 1,898 infertile woman (1,394 with oocyte maturation arrest and 504 with abnormalities in fertilization and early embryonic development). In addition, we also found other candidate pathogenic genes through bioinformatics analysis, but the pathogenicity of these mutant genes needs to be further verified by *in vitro* experiments and the generation of knockout or patient‐derived mutated animal models. Nevertheless, the genetic basis involved in the majority of affected individuals remain unknown, which requires extensive exploration using different research strategies. Previous studies have shown that gene‐based burden test is an effective strategy for finding novel pathogenic genes in many other disorders (Cirulli *et al*, 2015; Guo *et al*, 2018; Malik *et al*, 2021). By using the strategy, *LHX8* and *KPNA7* variants were identified to cause female infertility (Zhao *et al*, 2022; Wang *et al*, 2023). We believe that applying gene‐based burden test will reveal more genetic basis of female infertility. In addition, there is a good understanding of the functional impact of protein‐coding variants in female infertility (Jiao *et al*, 2021), but less understanding of variants in non‐coding regions, which accounts for 98% of the human genome. In the last decade, many studies have identified that variants in non‐coding regions cause different diseases (Turro *et al*, 2020; Wright *et al*, 2021; Wakeling *et al*, 2022), which suggests that the analysis of non‐coding regions in female infertility patients with unknown causes is also a powerful strategy to find novel pathogenic variants.

In conclusion, we identified bi‐allelic pathogenic variants in *PABPC1L* that cause oocyte maturation arrest and female infertility. Our findings provide direct evidence for the important role of PABPC1L in female reproduction and thus add a potential genetic candidate gene to be screened for causes of infertility patients in the clinic.

## Materials and Methods

### Clinical samples

In this study, five infertile individuals with pathogenic variants in *PABPC1L* and healthy controls were recruited from the Reproductive and Genetic Hospital of CITIC‐Xiangya, the Shanghai Ji Ai Genetics and IVF Institute affiliated with the Obstetrics and Gynecology Hospital of Fudan University, and the Reproductive Medicine Center of the Shaanxi Maternal and Child Care Service Center. A cohort of 504 infertile individuals with abnormalities in fertilization and early embryonic development was recruited from 62 collaborating hospitals. All blood samples were donated for the investigation after informed consent was obtained. The study was approved by the Ethics Committee of the Medical College of Fudan University and the Reproductive Study Ethics Committee of the hospitals. These experiments conformed to the principles set out in the WMA Declaration of Helsinki and the Department of Health and Human Services Belmont Report.

### Whole‐exome sequencing and bioinformatics analysis

Genomic DNA was extracted from peripheral blood using the QIAamp DNA Blood Mini Kit (Qiagen), and whole‐exome sequencing was performed using the SeqCap EZ Exome Kit (Roche) on an Illumina HiSeq 3000 platform (Illumina). Sequencing analysis was compared with the human reference sequence (NCBI Genome build GRCh37). Variants were annotated with GRCh37 and the dbSNP (version 138) database. All candidate variants were filtered using the following criteria: (1) variants that conformed to the autosomal recessive inheritance pattern; (2) variants with a minor allele frequency less than 0.1% (for homozygous variants) or 1% (for compound heterozygous variants) in the public databases, including gnomAD, 1000 Genomes Project, or ExAC databases, and not existing in our control database; (3) variants predicted to be loss of function or to be damaging by SIFT, PolyPhen‐2, and MutationTaster; and (4) variants within genes that were highly expressed or specifically expressed in oocytes. The candidate pathogenic variants were subsequently verified by Sanger sequencing of the affected probands as well as all the available family members. The primers used are shown in Appendix Table S1.

### *PABPC1L* expression analysis and real‐time quantitative PCR (qRT‐PCR)

Total RNA from control samples (including human GV, MI, and MII oocytes, Day 3 embryos (8‐cell), blastocysts, and other somatic tissues) were extracted with the RNeasy Mini Kit (Qiagen). Reverse transcription was performed with the PrimeScript RT Reagent Kit (Takara) according to the manufacturer's instructions. qRT‐PCR was performed with TB Green Premix Ex Taq (Takara) in triplicate on a LightCycler 480 II System (Roche). The expression of *PABPC1L* was normalized by comparison to the expression of an internal human *GAPDH* control. The qRT‐PCR primers for *PABPC1L* and *GAPDH* are shown in Appendix Table S1.

### Construction of expression plasmids and transfection

The full‐length coding sequence and 3′‐untranslated region (3′UTR) of *PABPC1L* was amplified from control human oocyte cDNA. The coding sequence product was then cloned into the pcDNA3 vector containing the N‐HA tag or the pCMV6‐Entry vector containing the C‐FLAG tag, and the 3′UTR product was cloned into the psiCHECK‐2 vector. Site‐directed mutagenesis was performed to introduce the identified variants (p.Gly97Asp, p.Arg176*, p.Ser137Phe, p.Ile320Asnfs*122, p.Arg501Trp, p.Arg374Gln, and c.*45G>C) into the wild‐type (WT) vector according to the instructions of the KOD‐Plus‐Mutagenesis Kit (Toyobo Life Science). HeLa cells (Cell Bank of Shanghai Institute for Biological Sciences) were cultured in DMEM (Gibco) supplemented with 10% fetal bovine serum (Gibco) and 1% penicillin/streptomycin (Gibco) in a 5% CO_2_ atmosphere at 37°C. The WT and mutant expression plasmids were transfected into HeLa cells using the PolyJet *In Vitro* DNA Transfection Reagent (Signagen) according to a standard protocol.

### Oligo(dT) pull‐down assay

HeLa cells were harvested 36 h post‐transfection and washed three times with cold PBS. Cells were lysed in NP‐40 lysis buffer with 1% protease inhibitor cocktail (Bimake) and RNase inhibitor (NEB). After quantification with the Bicinchoninic Acid Protein Assay Kit (Bo Cai Biological Technology), equal amounts of protein extracts were incubated with Oligo d(T)_25_ magnetic beads (NEB). After incubation at 4°C for 2 h, the beads were washed with lysis buffer three times. The bead‐bound proteins were eluted using sodium dodecyl sulfate (SDS) sample buffer.

### Immunoblot analysis

All immunoblot samples were heated at 100°C for 10 min. Equal amounts of protein were separated by SDS‐polyacrylamide gel electrophoresis (EZBiolab) and transferred to nitrocellulose membranes (Pall Corporation). The membranes were blocked with 5% non‐fat milk diluted in PBS with 0.1% Tween 20 (PBST) for 1 h and then incubated at 4°C overnight with primary antibodies. The membranes were washed with PBST three times and incubated with secondary antibodies for 1 h at room temperature followed by washing again with PBST three times. Finally, enhanced chemiluminescence imaging was performed on a chemiluminescent imaging system (5200, Tanon). Quantitation of immunoblot results was performed with the ImageJ software (National Institutes of Health). The following primary antibodies were used for immunoblotting: antibodies against HA Tag (3724, CST), HuR (2582, CST), and pERK1/2 (4370, CST) were used at 1:1,000 dilution, and antibodies against Vinculin (13901, CST) and Tubulin (T6199, Sigma) were used at 1:3,000 dilution as the internal controls. The secondary antibodies were goat anti‐rabbit IgG (1:5,000 dilution, M21001L, Abmart) or goat anti‐mouse IgG (1:5,000 dilution, M21002L, Abmart) conjugated to horseradish peroxidase.

### Immunofluorescent staining

HeLa cells were transfected with WT or mutant *PABPCL* expression plasmids, and cells were fixed for immunofluorescence after culturing for 36 h. Transfected cells were first treated with 4% paraformaldehyde (P6148, Sigma) for 30 min at room temperature, washed three times in PBS, and then blocked in a solution containing 1% bovine serum albumin (BSA) (B2064, Sigma) and 0.1% Triton X‐100 (T8787, Sigma) in PBS for 1 h at room temperature. The cells were washed again and then incubated with anti‐FLAG M2‐FITC antibody (1:200 dilution, F4049, Sigma) for 1 h at 37°C. After being washed three times, the cells were incubated with Hoechst (1:500 dilution, 33342, BD Biosciences) for 15 min at room temperature. Images were captured with the EVOS M7000 Microscope Imaging System (Thermo Fisher Scientific).

Mouse zygotes were fixed with 2% paraformaldehyde diluted in PBS for 30 min at room temperature and then permeabilized in PBS containing 0.5% Triton X‐100 for 20 min at room temperature. After incubation for 2 h in blocking buffer (3% BSA diluted in PBS with 0.1% Tween‐20 and 0.01% TritonX‐100) at room temperature, zygotes were stained with anti‐pERK1/2 (1:400, 4370, CST) primary antibodies diluted in blocking buffer for 1 h at 37°C. After three washes, samples were incubated with Alexa Fluor 488‐conjugated donkey anti‐rabbit (1:500, A21206, Invitrogen) secondary antibodies for 1 h at 37°C. Pronucleus was briefly stained with Hoechst (1:500, 33342, BD Biosciences) for 15 min at room temperature. The samples were in PBS and imaged using a laser scanning confocal microscopy (LSM880, Zeiss).

### Luciferase assay

The luciferase assay was performed with the Dual‐Luciferase Reporter Assay System (Promega). WT and mutant *PABPC1L* 3′UTR were cloned into the C‐terminus of the Renilla luciferase gene in the psiCHECK™‐2 vector. Empty vector was used as the control, and Firefly luciferase expression served as the internal standard for transfection efficiency. HeLa cell extracts were prepared 36 h after transfection and assayed for the Firefly and Renilla luciferase activities. Renilla luciferase activity values were normalized to Firefly activity. Results are presented as the means obtained from three independent experiments.

### Generation of *Pabpc1l* KI mice

*Pabpc1l* KI mice were generated using the CRISPR‐Cas9 gene‐targeting approach. All animal operations were approved by the Shanghai Medical College of Fudan University. The guide RNA (gRNA) target site for mouse *Pabpc1l* close to the disease variant was selected using the CRISPR design tool (G97D‐gRNA: 5′‐GGC CTT CGG AAG TCT GGT ATG GG‐3′, S137F‐gRNA‐1: 5′‐TAC AAT GAA CAT GGA TCG CGG GG‐3′, S137F‐gRNA‐2: 5′‐ATA CAA TGA ACA TGG ATC GCG GG‐3′, R374Q‐gRNA‐1: 5′‐ACA GCG CAA GGA GGA GCG GAA GG‐3′, R374Q‐gRNA‐2: 5′‐TGG CAC AGC GCA AGG AGG AGC GG‐3′). Additionally, the donor template of G97D and R374Q contained the synonymous AAG to AAA p.K95 mutation and GAG to GAA p.E373 mutation to avoid gRNA recognition and excision. To produce the Cas9 mRNA, gRNA, and donor oligo, a T7 promoter was first fused to the Cas9 coding region of the px330 plasmid by PCR amplification. The T7‐Cas9 PCR product was purified and used as a template for *in vitro* transcription using the mMESSAGE mMACHINE T7 ULTRA Kit (Life Technologies). A T7 promoter was fused to the gRNA template by PCR amplification, and the T7‐gRNA PCR product was purified and used as a template for *in vitro* transcription using the MEGAshortscript T7 Kit (Life Technologies). Both the Cas9 mRNA and the gRNAs were purified using a MEGAclear kit (Life Technologies) and eluted in RNase‐free water. Oligo donors were obtained as ultramer DNA oligos from GenScript Corporation. Female C57BL/6 mice (Beijing Vital River Laboratory Animal Technology Co.) were superovulated by injecting 10 IU of pregnant mare serum gonadotropin (PMSG, Ningbo Second Hormone Factory), followed 48 h later by injection of 10 IU of human chorionic gonadotropin (hCG, Ningbo Second Hormone Factory). They were then mated to C57BL/6 males, and fertilized embryos were collected from the oviducts. Cas9 mRNA (100 ng/ml), gRNAs (50 ng/ml), and donor oligos (100 ng/ml) were mixed and injected into the cytoplasm of fertilized oocytes using an IM300 microinjector (Narishige). About 30 injected single‐cell embryos were transferred into the oviducts of pseudopregnant ICR females at 0.5 days after coitus. For the identification of *Pabpc1l* point mutation founders, DNA was extracted and the target fragment was amplified followed by sequence analysis. The primers are shown in Appendix Table S1.

### Mouse oocyte collection, fertilization, and embryo culture *in vitro*

To obtain GV oocytes, ovaries were isolated from female mice (10–12 weeks old). GV oocytes were isolated from ovaries by puncturing the antral follicles with a fine needle on the stage of a dissecting microscope. For IVF, female mice (10–12 weeks old) were intraperitoneally injected with 10 IU of PMSG. After 46 h, mice were injected with 10 IU of hCG. After an additional 13 h, oocyte/cumulus masses were surgically isolated from the oviducts and were mixed with sperm in human tubal fluid medium (Nanjing Aibei Biotechnology Co.) for IVF. Zygotes were cultured in drops of K‐modified simplex optimized medium (Nanjing Aibei Biotechnology Co.) under oil at 37°C in an atmosphere of 5% CO_2_, and embryo development was monitored at regular intervals. All mouse experiments were reviewed and approved by the Shanghai Medical College of Fudan University.

### RNA sequencing (RNA‐seq)

Each sample included five zygotes. RNA‐seq libraries were prepared using the cDNA Synthesis, Amplification and Library Generation Kit (E6420, NEB). RNA quality was examined by gel electrophoresis and with Qubit (Thermo). RNA samples were sequenced on the Illumina Novaseq 6000 instrument by Genergy Biotechnology Co. Ltd. The raw data were handled by trim galore software, and data quality was checked by FastQC v0.11.2. The read length was 2 × 150 bp. Clean reads were aligned to the mouse genome mm10 using STAR and StringTie. The expression levels of the genes were quantified using normalized fragments per kilobase million (FPKM). Differential gene expression analysis was performed by DESeq2 with read counts data. The thresholds for determining differentially expressed transcripts were abs[Fold Change] > 1.5 and FDR < 0.05. Venn software was used to identify the co‐upregulated and co‐downregulated transcripts in the two databases (G97D vs. WT and S137F vs. WT). Gene Ontology (GO) term analysis was performed using the DAVID online tool. The expression of Mos‐MAPK pathway‐related genes and *Pabpc1l* were determined using qRT‐PCR with mouse *Actb* expression as the internal control. The primers are shown in Appendix Table S1.

### Statistical analyses

Experiments were performed with biological replicates as indicated in the Figure legends, and GraphPad Prism (GraphPad Software) was used to perform the statistical analyses. Differences were analyzed by Student's *t*‐tests when comparing experimental groups. Statistically significant values of *P* < 0.05, *P* < 0.01, *P* < 0.001, and *P* < 0.0001 are indicated by (*), (**), (***), and (****), respectively.

## Author contributions

**Lei Wang:** Conceptualization; supervision; funding acquisition; writing – review and editing. **Weijie Wang:** Conceptualization; data curation; formal analysis; funding acquisition; investigation; visualization; methodology; writing – original draft; writing – review and editing. **Jing Guo:** Resources; investigation. **Juanzi Shi:** Resources; investigation. **Qun Li:** Data curation; formal analysis. **Biaobang Chen:** Data curation; formal analysis. **Zhiqi Pan:** Formal analysis; investigation. **Ronggui Qu:** Investigation. **Jing Fu:** Resources; investigation. **Rong Shi:** Resources; investigation. **Xia Xue:** Resources; investigation. **Jian Mu:** Methodology. **Zhihua Zhang:** Formal analysis. **Tianyu Wu:** Visualization. **Wenjing Wang:** Methodology. **Lin Zhao:** Validation. **Qiaoli Li:** Project administration. **Lin He:** Resources. **Xiaoxi Sun:** Resources. **Qing Sang:** Conceptualization; supervision; funding acquisition. **Ge Lin:** Resources; supervision.

## Disclosure and competing interests statement

The authors declare no competing interests.

## For more information

- GenBank, https://www.ncbi.nlm.nih.gov/genbank/
- gnomAD, https://gnomad.broadinstitute.org/
- MutationTaster, http://mutationtaster.org/MutationTaster
- OMIM, https://www.omim.org
- PolyPhen‐2, http://genetics.bwh.harvard.edu/pph2/
- SIFT, https://sift.bii.a‐star.edu.sg
- DAVID online tool, https://david.ncifcrf.gov

## Supporting information



Appendix

Expanded View Figures PDF

Dataset EV1

Source Data for Expanded View

PDF+

Source Data for Figure 1

Source Data for Figure 2

Source Data for Figure 3

Source Data for Figure 4

Source Data for Figure 6

## Data Availability

The processing RNA‐seq dataset supporting conclusion is included within the supplemental data. The raw sequence data reported in this paper have been deposited in the Genome Sequence Archive in National Genomics Data Center, China National Center for Bioinformation/Beijing Institute of Genomics, Chinese Academy of Sciences (GSA: CRA009502) that are publicly accessible at https://bigd.big.ac.cn/gsa/browse/CRA009502.
